# Racing against time: Emergency ambulance dispatches and response times, a register-based study in Region Zealand, Denmark, 2013–2022

**DOI:** 10.1186/s13049-024-01284-0

**Published:** 2024-11-06

**Authors:** Josefine Tangen Jensen, Thea Palsgaard Møller, Stig Nikolaj Fasmer Blomberg, Annette Kjær Ersbøll, Helle Collatz Christensen

**Affiliations:** 1 Prehospital Center, Region Zealand, Ringstedgade 61, 13th floor, 4700 Næstved, Denmark; 2 Department of Clinical Medicine, University of Copenhagen, Copenhagen, Denmark; 3 Department of Anesthesiology and Intensive Care Medicine, Holbæk Hospital, Smedelundsgade 60, 4300 Holbæk, Denmark; 4 National Institute of Public Health, University of Southern Denmark, Copenhagen, Denmark; 5 Emergency Medical Services, Capital Region of Denmark, Ballerup, Denmark

**Keywords:** Emergency medical services, Ambulances, Emergency medical dispatch, Transportation of patients, Conveyance, Comorbidity

## Abstract

**Background:**

The global strain on Emergency Medical Services (EMS) is reflected in the increasing number of emergency ambulance dispatches. Shorter EMS response times have demonstrated some effect on very specific and rare conditions. It is unknown if the increased number of ambulance dispatches compromises response times.

This study aimed to describe trends in emergency ambulance dispatches and response times from 2013 to 2022 in Region Zealand, Denmark. Additionally, it aimed to outline the demographic profile of emergency ambulance patients, including age and comorbidities.

**Methods:**

Using administrative data from the Region Zealand emergency medical dispatch center, a register-based study spanning from January 1, 2013, to December 31, 2022, was conducted. Data were linked with nationwide registries and priority A (emergency), or B (urgent) ambulance dispatches were included.

Trends were examined overall and stratified by catchment areas corresponding to the hospitals with emergency departments in the region. Poisson and ordinal logistic regressions were used to analyze data.

**Results:**

The study encompassed 678,789 emergency ambulance dispatches, with 55.0% priority A and 45.0% priority B. Among these, 667,788 had a valid personal identification number allowing for further analysis. Within the study population, females comprised 48.5%, while 49.1% of patients were 65 years or more. Overall, 47.5% of patients had no comorbidities, while 7.7% and 44.8% had mild and severe comorbidities, respectively.

Emergency ambulance dispatches increased from 56,867 in 2013 to 81,080 in 2022 (143%). Correspondingly, the dispatch incidence rate per 1,000 residents per year increased from 69.2 to 95.5. Stratification by catchment areas revealed significant disparities.

The median response time for priority A dispatches increased from 7 min:14 s in 2013 to 8 min:20 s in 2022 and for priority B dispatches from 12 min:23 s to 15 min:6 s.

**Conclusions:**

From 2013 to 2022, emergency ambulance dispatches both priorities A and B increased in absolute numbers and per 1000 residents per year. Ambulance response times also increased for both priorities during the study period. The study shows regional disparities regarding to the rate of emergency ambulance dispatches and response times indicating challenges in resource distribution in the future for maintaining emergency care standards.

**Supplementary Information:**

The online version contains supplementary material available at 10.1186/s13049-024-01284-0.

## Background

The increasing demand for Emergency Medical Services (EMS) is a growing issue reflected by the increasing number of emergency ambulance dispatches. In the North Denmark Region, the number of patients conveyed to a hospital following a 1-1-2 emergency call increased by 67% between 2007 and 2014 corresponding to an increase from 24.3 to 40.2 patients per 1,000 residents per year [1]. Similar patterns have been observed internationally. An Australian study documented a 75% increase in rate of emergency ambulance transportations from 1995 to 2008 [2]. Furthermore, a Japanese study found a more than 20-fold increase in emergency dispatches from 1963 to 2010 indicating a continued increase despite a declining population [3].

Knowledge is crucial for decision makers regarding what is driving the increasing demand. Demographic changes, particularly the ageing population, appear to play a significant role. An Australian study found that patients older than 85 years disproportionately drove the increasing usage of emergency ambulances [2]. In Denmark, the proportion of residents aged 60 years or more conveyed by ambulance increased from 39.9% in 2007 to 48.6% in 2014 [1]. Population projections suggest a further increase as the population continues to age. The proportion of Danish residents aged 65 years or more is expected to increase from 20% in 2023 to 25% in 2040 and in parts of the country, this number is even higher [4].

Another potential driver of the increasing EMS demand seems to be comorbidities, but knowledge is limited. One Danish study examining emergency ambulance patients found that the proportion of patients with high comorbidity (Charlson Comorbidity Index (CCI) ≥ 3) increased from 6.4 to 9.4% during an 8-year study period (2007–2014) [1]. Another Danish study found that repeated use of ambulance services is also associated with CCI ≥ 3 [5].

Altogether, there is a knowledge gap, both when it comes to drivers for the increasing ambulance demand, and in terms of how the demand affects EMS response times. Given the crucial role of EMS response times in managing some acute medical conditions such as out-of-hospital cardiac arrest [6–8], myocardial infarction [9], and stroke [10–12], it is important to understand how the increasing demand for EMS impacts service efficiency. To strengthen the EMS in the future it is necessary to know more about the present situation and the development over the past decade.

The primary aim of the study was to investigate trends in the number of emergency ambulance dispatches and response times during 10 years (2013–2022) in Region Zealand, Denmark. The secondary aim was to evaluate the distribution of age and comorbidity of the patients requiring an emergency ambulance. The aims were studied overall and stratified by catchment areas corresponding to the regional hospitals with emergency departments.

## Methods

### Design

A register-based study of all emergency ambulance dispatches in Region Zealand from 2013 to 2022 was undertaken. The study adhered to the RECORD statement [13], an extension of the STROBE statement.

### Setting

Denmark has a population of 5.9 million (January 2022) [4]. Since 2007, the country has been divided into five administrative regions, each responsible for their own healthcare system including EMS. Medical assistance and admission to hospitals are free of charge for residents. For non-emergencies general practitioners are available during working hours. On weekends, holidays and outside of working hours an out-of-hours doctor’s service is available, where patients can be given advice on the telephone or be examined in person. The EMS are coordinated through a network of regional Emergency Medical Dispatch Centers (EMDCs). When someone calls the emergency number 1-1-2, trained dispatchers (specially trained nurses or paramedics) assess the situation, and dispatch appropriate resources, including ambulances, and if needed, advanced emergency response units. The dispatcher’s decisions are supported by a criteria-based dispatch protocol called Danish Index for Emergency Care [14]. A new administrative EMDC software was introduced in October 2017, but the dispatch criteria have remained the same throughout the study period.

Determined by law, ambulances must be staffed with at least two emergency medical technicians but can also be staffed with paramedics [15]. The advanced emergency response units comprise a helicopter emergency medical service and mobile critical care units staffed with physicians and paramedics [14].

**Fig. 1:**
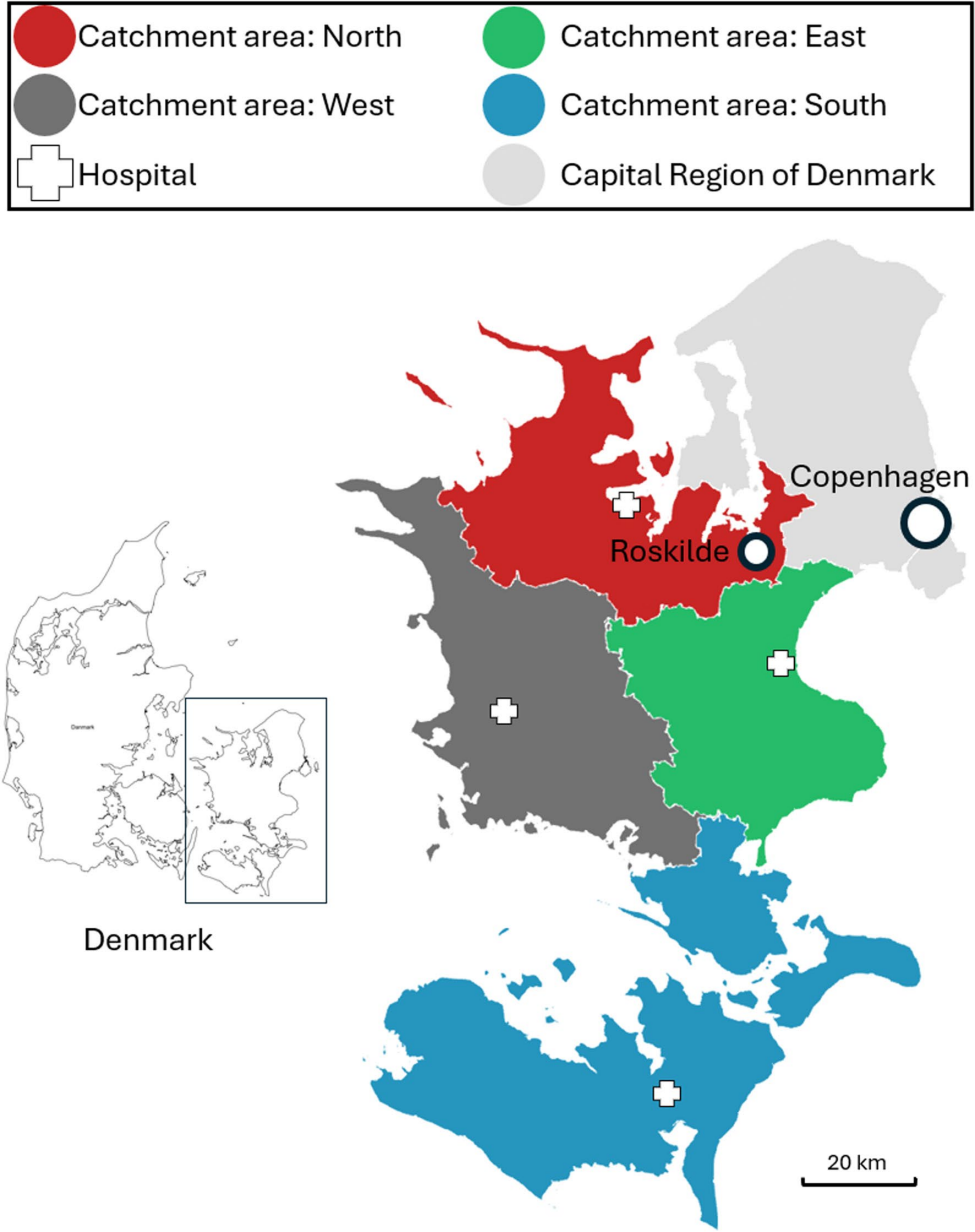
Map of Region Zealand. Illustrating the four catchment areas corresponding to the four hospitals with emergency departments in the region

Region Zealand is in the eastern part of the country covering an area of 7274 km^2^ with a total of 850,000 residents. The largest city is Roskilde with 51,916 residents and there are four other cities with 20,000–45,000 residents [4]. As of 2022, the region is divided into four catchment areas each corresponding to a hospital with an emergency department (Fig. 1). Two emergency departments have been closed during the study period. Region Zealand has the lowest life expectancy compared to the other regions in Denmark [4].

A personal identification number is assigned to all Danish residents at birth or immigration. This allows for individual-level linkage of data to all Danish registries [16].

### Participants

All emergency ambulance dispatches in Region Zealand from January 1, 2013 to December 31, 2022 were eligible for inclusion. An emergency ambulance dispatch is categorized as either priority A or B. Priority A with lights and sirens is sent to patients with potential life-threatening conditions, and priority B without lights and sirens is sent to patients with conditions that could evolve to be potentially life-threatening. An ambulance can be dispatched either because of a 1-1-2 call or if healthcare professionals request it directly (general practitioners, pre- or in-hospital personnel). There are two other priority types in the Danish EMS: Priority C, an urgent or planned transport for patients requiring monitoring and possibly treatment during transport and priority D, a planned supine transport for patients not requiring monitoring or treatment.

Interhospital ambulance transports and ambulance dispatches to incidents outside the region were excluded. When patients had more than one separate ambulance transport during the study period, all their dispatches were included. If more than one ambulance was sent per patient per incident, only one ambulance was included in the study. Ambulance dispatches were included for patients not conveyed to a hospital. Response time was calculated from the request of an ambulance until arrival of the first ambulance at the scene. Ambulance dispatches with negative response times were excluded from response time analyses.

For comparison, absolute numbers were also calculated for ambulance dispatches, priorities C and D. In addition to exclusion criteria mentioned above, dispatches were also excluded if the patient was conveyed away from a hospital, for example to their home. It was not possible to identify dispatches where the patient was conveyed to a hospital for ambulatory care.

### Data sources

Data were obtained from administrative data routinely collected by the EMDC in Region Zealand. The database used to create the study population contained all EMS dispatches in the study period. Due to a change in the administrative EMDC software in October 2017, the final dataset covering the total study period from 2013 to 2022 was constructed from two different datasets, which had a dissimilar data structure.

The datasets were managed independently, and the variables of interest were adjusted to ensure uniformity in the final dataset used for analyses. Data contained extensive information regarding each ambulance dispatch including patient’s personal identification number, time stamps and geographical information. To calculate the Charlson Comorbidity Index (CCI) [17], diagnoses (For the ICD-codes used to calculate CCI, see Additional file 1) were obtained from the Danish National Patient Register [18] and linked according to the patient’s personal identification number. CCI was calculated based on data from a 10-year period prior to the emergency ambulance dispatch to cover each patient’s comorbidity diagnoses thoroughly. CCI was grouped into 0 (no comorbidity), 1–2 (mild comorbidity) and ≥ 3 (severe comorbidity). CCI was set to 0 for patients with none of the relevant diagnoses in the Danish National Patient Register.

Data were securely stored according to applicable guidelines.

### Variables

The primary outcomes were absolute number of emergency ambulance dispatches, incidence rate (IR) of ambulance dispatches per 1000 residents per year and median response time. The secondary outcomes were sex, age group and CCI of the patients. Age and sex were derived from the patient’s personal identification number.

### Statistical analysis

A descriptive analysis of characteristics of the study population was performed by means of frequency distributions (n, %) overall and stratified by catchment area.

Poisson regression analyses were used to investigate time trends in emergency ambulances dispatches per 1,000 residents per year. The analyses included calendar year, and catchment area as independent variables. The analyses were adjusted for the median age of the ambulance patients to account for an increasing age of the patients. The reference year was 2013 to compare later changes, and the reference catchment area was “East”, which had the lowest incidence rate. Associations were presented as incidence rate ratios (IRR) with corresponding 95% confidence intervals (95% CI).

Ordinal logistic regression analyses were used to evaluate trends in the odds of higher comorbidity. The analyses included calendar year, catchment area and age group (< 50, 50–59, 60–69, 70–79, ≥ 80 years) as independent variables. Associations were presented as odds ratio (OR) with corresponding 95% CI.

Changes in response time for priorities A and B, respectively, were analyzed using linear regression with calendar year and catchment area as the independent variables. Because the response time was left-skewed, both log- and rank-transformations were applied.

All analyses were performed overall and stratified by catchment area (East, North, South, West) where the incident occurred. A *p*-value < 0.05 was considered statistically significant.

Data management and statistical analysis were performed using R 4.4.0.

## Results

**Fig. 2:**
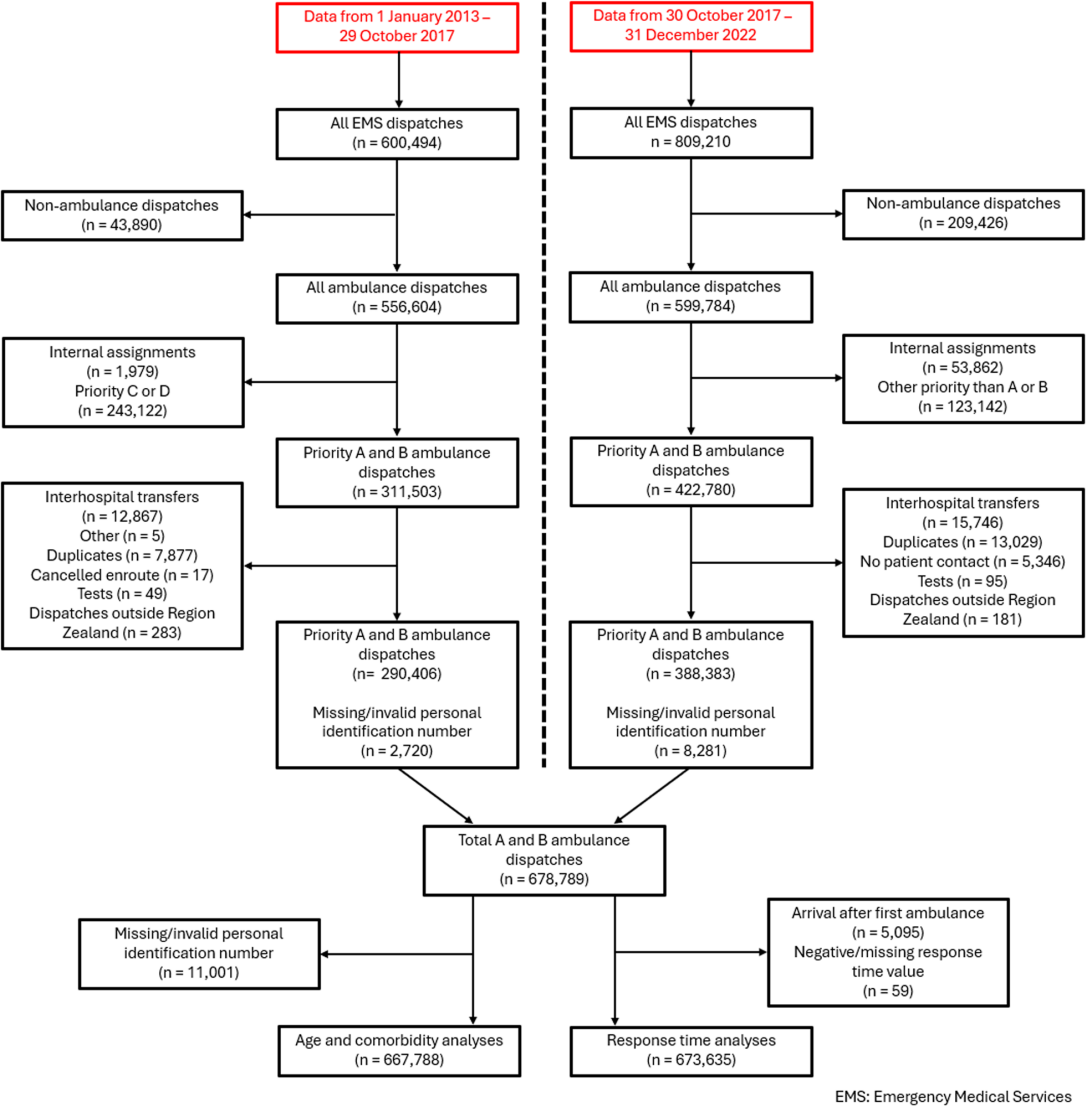
Data flowchart

In the study period, 678,789 emergency ambulance dispatches were included (Fig. 2). The overall distribution between ambulance priorities A and B was 55.0% and 45.0%, respectively. Of these, 667,778 patients had a valid personal identification number, and 605,857 patients had a registered diagnosis in the Danish National Patient Register for calculating CCI. Within the study population, females comprised 48.5%, while most patients (49.1%) were in the age group of 65 years or more. Additionally, 47.5% had no comorbidities, 7.7% had mild comorbidities, and 44.8% had severe comorbidities. See Table 1 for characteristics of the patient population.

**Table 1:** Characteristics of patients included in the study period 2013–2022

			Catchment area							
			Corresponding to the four hospitals with emergency departments in the region							
	Overall		East		North		South		West	
	678 789		167 906	(24.7)	173 365	(25.5)	154 982	(22.8)	182 536	**(27.3)**
**Sex**										
Female, n (%)	324 106	(48.5)	81 184	(49.1)	82 996	(48.7)	72 112	(47.3)	87 814	(48.8)
Male, n (%)	343 682	(51.5)	84 044	(50.9)	87 300	(51.3)	80 247	(52.7)	92 091	(51.2)
**Age group**										
0–14 years, n (%)	30 462	(4.6)	9 021	(5.5)	7 532	(4.4)	5 618	(3.7)	8 291	(4.6)
15–24 years, n (%)	51 844	(7.8)	12 000	(7.3)	14 322	(8.4)	10 422	(6.8)	15 100	(8.4)
25–64 years, n (%)	257 567	(38.6)	62 571	(37.9)	64 309	(37.8)	58 694	(38.5)	71 993	(40.0)
≥ 65 years, n (%)	327 915	(49.1)	81 636	(49.4)	84 133	(49.4)	77 625	(50.9)	84 521	(47.0)
**Charlsons Comorbidity Index**										
0 (no comorbidity), n (%)	287 613	(47.5)	73 751	(49.3)	77 325	(50.2)	61 158	(44.3)	75 379	(46.0)
1–2 (mild comorbidity), n (%)	46 527	(7.7)	10 786	(7.2)	10 849	(7.0)	10 229	(7.4)	14 663	(8.9)
≥ 3 (severe comorbidity), n (%)	271 717	(44.8)	65 110	(43.5)	65 960	(42.8)	66 791	(48.3)	73 856	(45.1)
*(missing, n)*	*11 001*		*2 678*		*3 069*		*2 623*		*2 631*	

n = 678 789 emergency ambulance dispatches

**Fig. 3:**
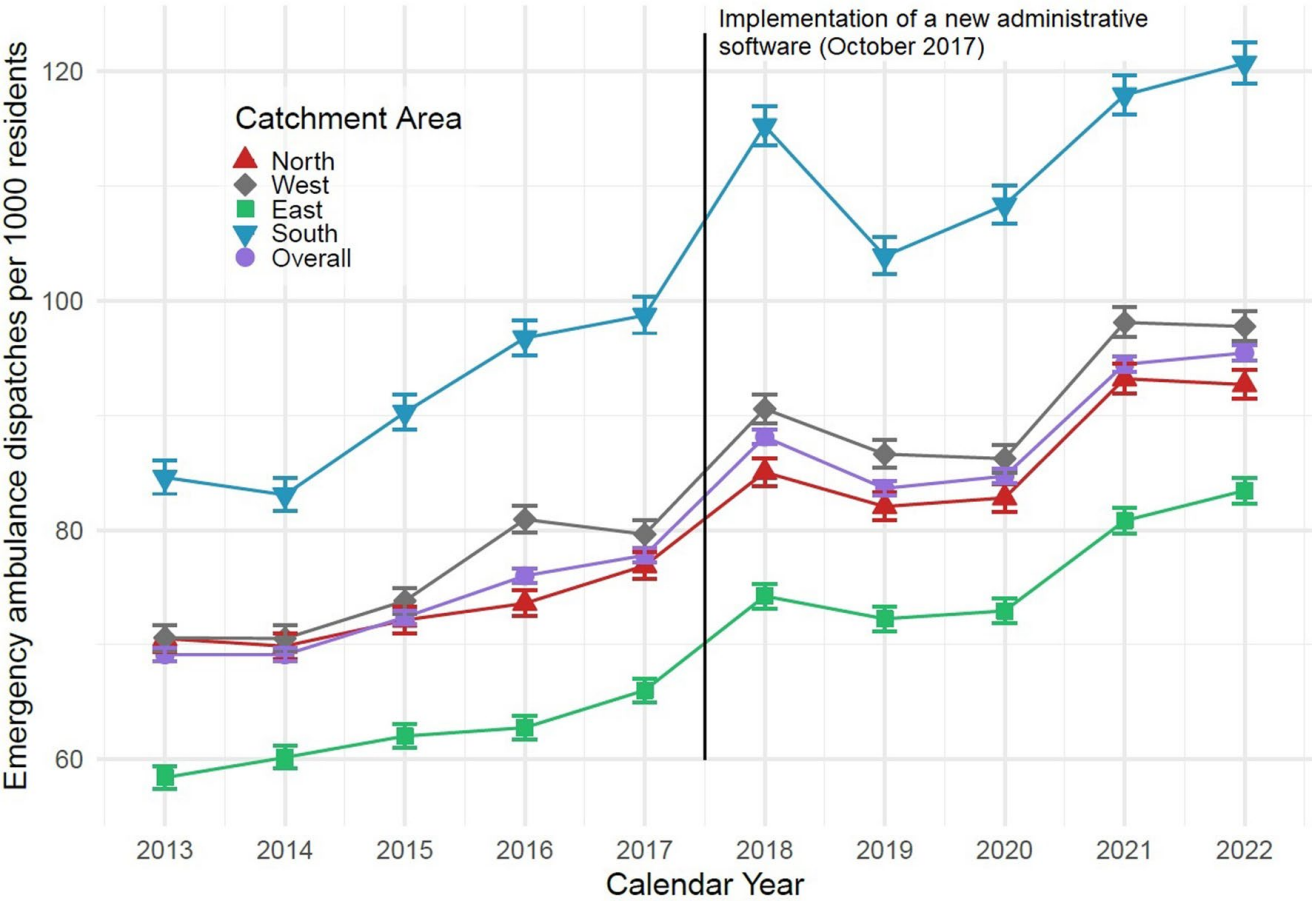
Distribution of emergency ambulance dispatches per 1,000 residents per year, 2013–2022. Data presented overall and stratified by catchment area corresponding to the four hospitals with emergency departments in the region

During the study period, annual emergency ambulance dispatches increased by 43% from 56,809 in 2013 to 81,048 in 2022. Correspondingly, the incidence rate increased from 69.1 [95% Confidence Interval (CI) 68.6; 69.7] to 95.4 [95% CI 94.8; 96.1] emergency ambulance dispatches per 1,000 residents per year (Fig. 3). The incidence rate of emergency ambulance dispatches was 35% higher in 2022 than in 2013 (IRR = 1.35 [95% CI 1.32; 1.39].

When including priorities C and D ambulance dispatches transporting patients to a hospital the total amount of annual dispatches increased by 18% from 76,954 in 2013 to 91,020 in 2022 (Table 2). The distribution between all priorities in 2013 was 45% priority A, 29% priority B, 23% priority C and 3% priority D. In 2022 the numbers were 47% priority A, 42% priority B, 7% priority C and 3% priority D. Priority C dispatches decreased by 62% from 17,751 in 2013 to 6,794 in 2022.

**Table 2:** Differences between 2013 and 2022

	Year	Overall		Catchment area							
				Corresponding to the four hospitals with emergency departments in the region							
				East		North		South		West	
Emergency ambulance dispatches, total	2013	56 809		13 659		14 870		13 096		15 184	
	2022	81 048	143%	20 783	152%	20 619	139%	18 029	138%	21 617	142%
Emergency ambulance dispatches, priority A	2013	34 300		8 263		8 866		8 059		9 112	
	2022	42 488	124%	11 027	133%	10 844	122%	9 369	116%	11 248	123%
Emergency ambulance dispatches, priority B	2013	22 509		5 396		6 004		5 037		6 072	
	2022	38 560	171%	9 756	181%	9 775	163%	8 660	172%	10 369	171%
Ambulance dispatches, priority C	2013	17 751		4 154		4 438		3 901		5 258	
	2022	6 794	38%	1 604	37%	1 710	37%	1 598	41%	1 882	36%
Ambulance dispatches, priority D	2013	2 394		474		615		472		833	
	2022	3 178	133%	754	159%	782	127%	891	189%	751	90%
Total ambulance dispatches, priority A-B-C-D	2013	76 954		18 287		19 923		17 469		21 275	
	2022	91 020	118%	23 141	127%	23 111	116%	20 518	117%	24 250	114%
Emergency ambulance dispatch rate per 1000 residents per year, total	2013	69.1		58.4		70.5		84.6		70.6	
	2022	95.4		83.4		92.7		120.7		97.8	
Emergency ambulance dispatch rate per 1000 residents per year, priority A	2013	41.7		35.3		42.1		52.1		42.4	
	2022	50.0		44.3		48.7		62.7		50.9	
Emergency ambulance dispatch rate per 1000 residents per year, priority B	2013	27.4		23.1		28.5		32.5		28.2	
	2022	45.4		39.2		43.9		58.0		46.9	
Patients aged ≥ 65 years,	2013	45.7%		46.7%		47.0%		46.3%		42.9%	
% of total emergency dispatches											
	2022	51.4%		51.0%		51.3%		54.4%		49.5%	
Patients with severe comorbidity (CCI ≥ 3)	2014	34.0%		31.8%		33.3%		36.3%		35.0%	
% of total emergency dispatches	2022	47.4%		46.1%		44.7%		51.9%		47.6%	
Residents aged ≥ 65 years in Region Zealand	2013	20.0%		18.5%		19.5%		24.0%		19.4%	
	2022	23.4%		21.3%		23.0%		28.6%		22.9%	

Data presented overall and stratified by catchment area corresponding to the four hospitals with emergency departments in the region. CCI: Charlson Comorbidity Index

Patients aged 65 years or more constituted 45.7% of the emergency ambulance patients in 2013 increasing to 51.4% in 2022. Regarding CCI, the proportion of patients with CCI ≥ 3 increased from 34.0 to 47.4%. The odds ratio (OR) of being in a more severe comorbidity group in 2022 compared to 2013 was 1.90 [95% CI 1.85; 1.95] overall.

**Fig. 4:**
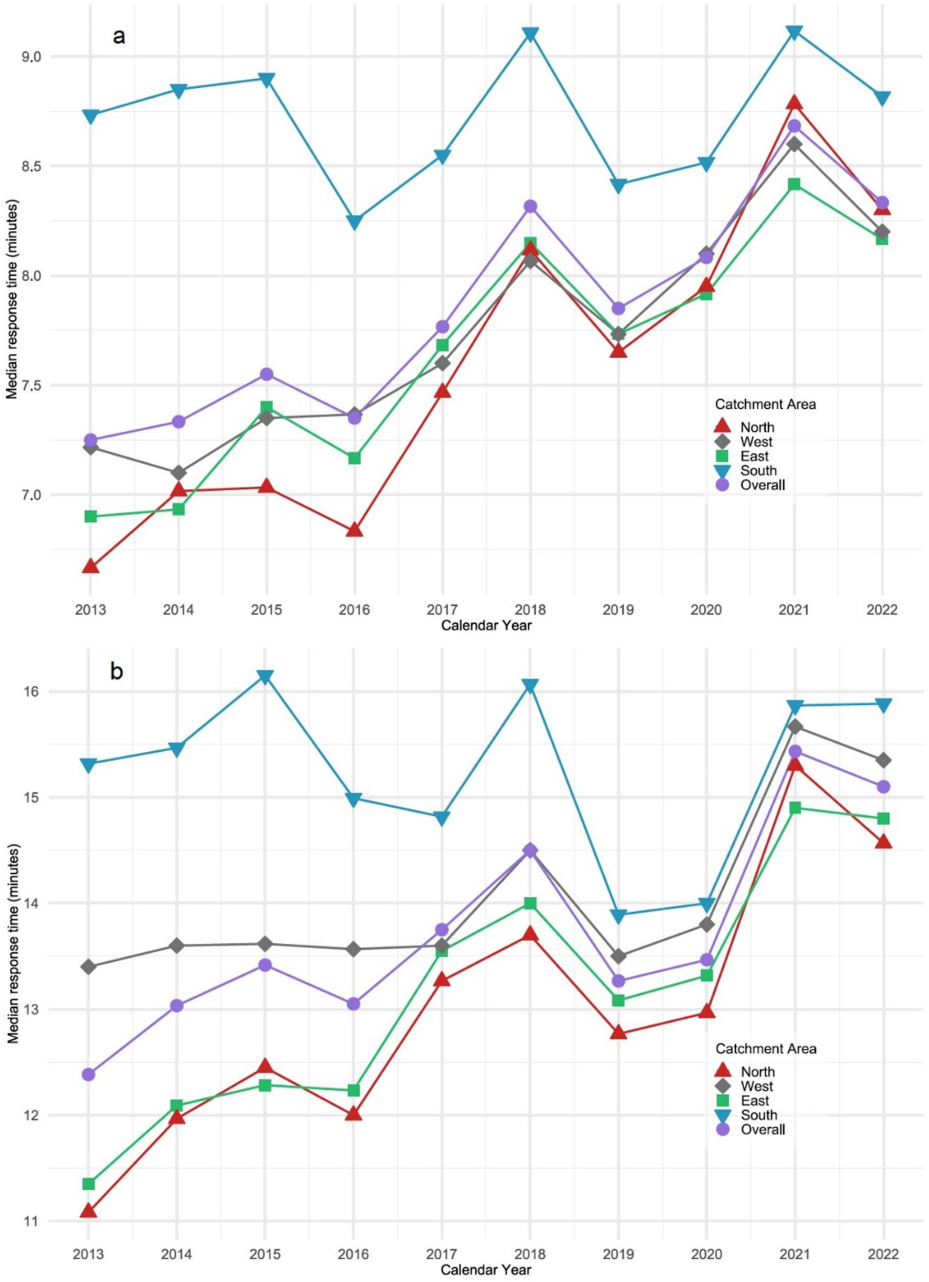
Distribution of median response time per year for priority A and B ambulances, 2013–2022. Data presented overall and stratified by catchment area corresponding to the four hospitals with emergency departments in the region. **a**. Priority A ambulances. **b**. Priority B ambulances

A total of 673,635 emergency ambulance dispatches were eligible for analyses of response times. In 2013, the median response time for emergency ambulance dispatches with priority A (Fig. 4a) was 7 min:15 secs, which increased to 8 min:20 secs in 2022. The median priority B response time (Fig. 4b) was 12 min:23 secs in 2013 and increased to 15 min:6 secs in 2022. Linear regression of response time (after log-transformation) showed a significant increase in response times for both priorities A and B from 2013 to 2022.

In the analyses stratified by catchment areas (Table 2) the same trends as in the overall results were seen, but there was a discrepancy regarding the southern catchment area. In this area, the rate of emergency ambulance dispatches per 1,000 residents per year was higher than in the other catchment areas (84.6 in 2013 and 120.7 in 2022). Across the ten-year study period, the IRR was 1.45 [95% CI 1.43; 1.46] in the southern catchment area compared to the eastern catchment area. There was also a higher proportion of older patients (54.4% in 2022) and patients with severe comorbidities (51.9% in 2022). The OR for more severe comorbidity in the southern area across the ten-year study period was 1.20 [95% CI 1.18; 1.22] and for 2022 was 1.98 [95% CI 1.88; 2.08]. Notably, while response times for both priorities A and B ambulance dispatches remained consistent in the southern catchment area, they increased in the three other catchment areas.

## Discussion

### Key results

During the decade from 2013 to 2022, the present study found an increase in emergency ambulance dispatches, both in absolute numbers and per 1,000 residents per year. This trend was observed across Region Zealand. The response times for priority A and B ambulances increased throughout the region. However, one of the four catchment areas maintained consistent response times, while an increase was found in the other three areas. The demographic characteristics of patients requiring an emergency ambulance changed over the study period. There was an increase in both the proportion of patients aged 65 or more as well as the proportion of patients with CCI ≥ 3.

The results regarding the increase in emergency ambulance dispatches are consistent with the trends observed in other studies [1–3]. The findings of increasing patient age also concur with these studies [1, 2] and the same applies to the increase in patients with CCI ≥ 3 [1].

The increasing number of emergency ambulance dispatches and the accompanying transformation in the patient demographics is a major task for the public health services and the EMS. Danish population age projections indicate that the increasing age trend seems to continue [4] and at the same time an accompanying increase in comorbidities can result in the ambulance crew having to assess more patients with a higher complexity. Consequently, on-scene times are expected to increase as shown in previous studies [19, 20], necessitating a need for more ambulances due to relatively longer periods of inaccessibility per patient [21]. High comorbidity has also been associated with repeated ambulance use [5] and the increasing OR of having a higher CCI could be a driver for the increasing numbers in the present study.

Similarly, emergency department crowding has been attributed to an increase in presentations by elderly patients and those with urgent and complex care needs [22]. Another factor contributing to the increasing emergency ambulance use could be patients utilizing EMS despite their condition perhaps being more appropriately managed by primary care providers. These cases are associated with increasing age and the lack of availability of primary care [23]. This decreased availability could be a factor in Region Zealand, where the number of general practitioners has declined by 17% during the study period [24]. Also supporting this hypothesis is the decline in the number of priority C dispatches, which are rarely dispatched following a 1-1-2 emergency call but rather after the patient has been assessed by a physician. In Australia, campaigns with a focus on patients’ perception of urgency have been initiated to try to affect the number of emergency calls [25].

In Region Zealand, response times are used as a quality measure and have been contractually agreed on between Region Zealand and the ambulance provider. A possible factor that might influence response times could be the number of available ambulances, but this data is not available. The location of ambulance stations and the dispatch criteria for emergency ambulances have remained the same throughout the study period. Regarding the influence of the COVID-19 pandemic, a Danish study has found that response times were unaffected [26]. It is unknown what can explain the fluctuations seen in this study and why the response time remained consistent in the southern catchment area, and it is an area of interest for further study to determine which factors affect response times.

Previous studies have shown that shorter response times are crucial when regarding out-of-hospital cardiac arrest [6–8], myocardial infarction [9] and stroke [10–12] but are not associated with mortality in trauma patients [8, 27, 28]. Even though response times increased during the study period, improving these may not be a solution to improve overall patient outcomes, because the diagnoses where shorter response times are important ultimately represent only a smaller proportion of the patients that are dispatched an emergency ambulance.

In Denmark, there is a social and geographical inequality concerning primary health care [29]. Examining geographical differences in this study, the southern catchment area was found to be different from the rest of the region regarding emergency ambulance dispatch rates, age composition, comorbidity distribution and the changes in response times. The dispatch rate was higher, the patients were older and had more comorbidity, but the response times remained consistent. Furthermore, an increased mortality in the southern catchment area compared to the rest of Denmark has been found [30]. This underlines the complexity of planning the EMS even within such a small geographical area as Region Zealand.

### Limitations

The study data were derived from two different databases. The registration of dispatches was different in the two databases decreasing the uniformity of the data. The amount of missing personal identification numbers was low (1.6%) but can possibly have entailed selection bias affecting the distribution of sex, age and CCI. The data for the calculation of CCI have improved over the study period [31], so that the patients may seem to have more comorbidity in the latter years possibly introducing temporal bias. On the other hand, equal access to EMS in Denmark limits the risk for selection bias due to differentiated inclusion. The results from this study have a high degree of generalizability in a Danish context [32]. The increasing amount of elderly is a global challenge [33] and this will continue to increase the burden on the EMS.

### Conclusions

From 2013 to 2022, emergency ambulance dispatches increased both in absolute numbers and per 1000 residents per year and at the same time, ambulance response times increased. Demographically, the ambulance population increased in age and comorbidity score. The study findings showed regional disparities according to both ambulance rates, response times and demographics.

The increasing age trend and higher comorbidity fuels the complexity of ambulance transportations. The results from this study indicate challenges in resource distribution in the future for maintaining emergency care standards, which is exacerbated by the changing demographics of the emergency ambulance patient population.

## Supplementary Information

Additional file 1.

## Data Availability

The datasets used and analyzed during the current study are not publicly available. Data are available from the corresponding author upon reasonable request with permission from the Regional Research Register, Region Zealand.

## Abbreviations

CCI: Charlson comorbidity index
CI: Confidence interval
EMDC: Emergency medical dispatch center
EMS: Emergency medical services
IR: Incidence rate
IRR: Incidence rate ratio
OR: Odds ratio
