# Supplementary material for: Racing against time: Emergency ambulance dispatches and response times, a register-based study in Region Zealand, Denmark, 2013–2022
Source: Scand J Trauma Resusc Emerg Med. 2024 Nov 6;32:108. doi: 10.1186/s13049-024-01284-0 (PMC11542390; doi:10.1186/s13049-024-01284-0)
Supplement: Supplementary file 1 — Additional file 1. [file 13049_2024_1284_MOESM1_ESM.pdf]

ICD-10 codes searched for in Danish Patient Registry to calculate Charlson Comorbidity Index

Disease category	ICD-10 codes	Weight	Number of codes
Myocardial infarction	DI21*, DI22*,DI23*	1	3
Congestive heart failure	DI50*, DI110, DI130, DI132	1	4
Peripheral vascular disease	DI70*, DI71*, DI72*, DI73*, DI74*, DI77*	1	6
Cerebrovascular accident or transient ischemic attack	DG45*, DG46*, DI60*, DI61*, DI62*, DI63*, DI64*, DI65*, DI66*, DI67*, DI68*, DI69*	1	12
Dementia	DF00*, DF01*, DF02*, DF03*, DG30*, DF051	1	6
Lung disease	DJ40*, DJ41*, DJ42*, DJ43*, DJ44*, DJ45*, DJ46*, DJ47*, DJ60*, DJ61*, DJ62*, DJ63*, DJ64*, DJ65*, DJ66*, DJ67*, DJ684*, DJ701, DJ703, DJ841*, DJ920, DJ961, DJ982*, DJ983	1	24
Connective tissue disease	DM05*, DM06*, DM08*, DM09*, DM30*, DM31*, DM32*, DM33*, DM34*, DM35*, DM36*, DD86*	1	12
Peptic ulcer disease	DK25*, DK26*, DK27*, DK28*, DK221*	1	5
Hemiplegia, paraplegia and tetraplegia	DG81*, DG82*	2	2
Kidney disease	DI12, DI13, DN00, DN01*, DN02*, DN03*, DN04*, DN05*, DN07*, DN11*, DN14*, DN17*, DN18*, DN19*, DQ61*	2	15
Cancer	DC0*, DC1*, DC2*, DC3*, DC4*, DC5*, DC6*, DC70*, DC71*, DC72*, DC73*, DC74*, DC75*, DC81*, DC82*, DC83*, DC84*, DC85*, DC88*, DC90*, DC91*, DC92*, DC93*, DC94*, DC95*, DC96*	2	26
Metastatic carcinoma	DC76*, DC77*, DC78*, DC79*, DC80*	6	5
Diabetes without complications	DE100, DE101, DE109*, DE110, DE111, DE119*	1	6
Diabetes with complications	DE102, DE103, DE104, DE105*, DE106, DE107, DE108, DE112, DE113, DE114, DE115*, DE116, DE117, DE118	2	14
Liver disease, mild	DB18*, DK71*, DK73*, DK74*, DK700, DK701, DK702, DK703*, DK709, DK760*	1	10
Liver disease, moderate to severe	DB150, DB160, DB162, DB190, DI85*, DK72*, DK704*, DK766*	3	8
AIDS & HIV	DB21*, DB22*, DB23*, DB24*	6	4

*Including all underlying codes
